# Differentiation of Closely Related Oak-Associated Gram-Negative Bacteria by Label-Free Surface Enhanced Raman Spectroscopy (SERS)

**DOI:** 10.3390/microorganisms9091969

**Published:** 2021-09-16

**Authors:** Dorotėja Vaitiekūnaitė, Valentinas Snitka

**Affiliations:** 1 Laboratory of Forest Plant Biotechnology, Institute of Forestry, Lithuanian Research Centre for Agriculture and Forestry, Liepų Str. 1, Girionys, 53101 Kaunas, Lithuania; 2 Research Center for Microsystems and Nanotechnology, Kaunas University of Technology, Studentu Str. 65, 51369 Kaunas, Lithuania; vsnitka@ktu.lt

**Keywords:** plant-associated bacteria, surface enhanced Raman spectroscopy, SERS, label-free, *Paenibacillus*, *Pseudomonas*, *Pantoea*, principal component analysis, discriminant function analysis

## Abstract

Due to the harmful effects of chemical fertilizers and pesticides, the need for an eco-friendly solution to improve soil fertility has become a necessity, thus microbial biofertilizer research is on the rise. Plant endophytic bacteria inhabiting internal tissues represent a novel niche for research into new biofertilizer strains. However, the number of species and strains that need to be differentiated and identified to facilitate faster screening in future plant-bacteria interaction studies, is enormous. Surface enhanced Raman spectroscopy (SERS) may provide a platform for bacterial discrimination and identification, which, compared with the traditional methods, is relatively rapid, uncomplicated and ensures high specificity. In this study, we attempted to differentiate 18 bacterial isolates from two oaks via morphological, physiological, biochemical tests and SERS spectra analysis. Previous *16S rRNA* gene fragment sequencing showed that three isolates belong to *Paenibacillus*, 3—to *Pantoea* and 12—to *Pseudomonas* genera. Additional tests were not able to further sort these bacteria into strain-specific groups. However, the obtained label-free SERS bacterial spectra along with the high-accuracy principal component (PCA) and discriminant function analyses (DFA) demonstrated the possibility to differentiate these bacteria into variant strains. Furthermore, we collected information about the biochemical characteristics of selected isolates. The results of this study suggest a promising application of SERS in combination with PCA/DFA as a rapid, non-expensive and sensitive method for the detection and identification of plant-associated bacteria.

## 1. Introduction

One of the limitations in microbiological research stems from the inability to easily differentiate bacterial samples at the species and/or strain level [1,2,3,4,5]. Over the years, many methods have been developed for microbial identification: morphological assessment, analytical profile index (API), immuno-assays (ex. ELISA), DNA sequencing, etc. The current “gold” standard, DNA sequencing, allows such discrimination based on the minute differences in bacterial genetic code, however, this method can be time consuming and economically inefficient, hence situationally ineffective [2,6,7,8,9,10,11,12,13,14].

Vibrational spectroscopy is a technique that has been used for the analysis of various chemicals, and in recent years has been successfully adapted for microbial research, showing great promise in becoming a novel diagnostic system in this field [4,5,6,8,9,15,16,17,18].

This technique stems from the fact that under excitation by light, analyte molecules will experience observable photon scattering. Raman scattering happens when the excitation energy is not the same as that of the scattered energy post interaction with the analyte molecule. Due to the low emission rate of scattered photons, an integration time of minutes is required to obtain useful spectra, however, ways to enhance this intrinsic aspect have been developed [18,19].

Raman spectroscopy, and its more sensitive variant (offering enhancements of up to 10^15^-fold [8,16,20,21])—surface enhanced Raman spectroscopy (SERS), are effective ways to discern bacterial samples up to strain level based on qualitative differences in cell chemistry [6,8,9,10,15,16,22]. This is sometimes referred to as vibrational “fingerprinting” or “barcoding”, as each organism presents different and unique spectra [4,23,24].

The sensitivity of the SERS technique is facilitated by substrate surface modifications. Analyte bioparticles are adsorbed on or placed in close proximity to a noble metal like silver (Ag) or gold (Au), nanoparticle (NP)-covered surfaces. In a sense this creates “hot spots”, which focus and thus strengthen the signal that is emitted by the analyte molecules under excitation [14,16,20,22,25]. This allows for shorter integration times and increased sensitivity compared to standard Raman spectroscopy. SERS is advantageous due to its potential as a low cost, rapid, low volume, non-destructive, broad information content, high specificity and sensitivity diagnostic method [1,9,10,18,20,22], also in part because analysis is easily performed in aqueous environments without interface [4,25]. In bacteriology, the efficacy of SERS was demonstrated with purified cultures, mixed and even single cell samples [6,7,8,9,13,15,20].

Spectra generated by SERS, provide the ability to carry out multivariate cluster analysis (ex. principal component analysis (PCA), discriminant function analysis (DFA), hierarchical cluster analysis (HCA), etc. or a combination thereof) that help the separation of different bacterial strains by determining both unique and common aspects of given spectra [8,9,15,16,22]. Moreover, these spectra help inform about the cell’s molecular structure and composition, because spectral features correspond to functional groups [6,7,20,22,26].

Despite the aforementioned advancements, the field is generally geared towards food and medical pathogen identification [1,2,6,7,8,9,10,12,14,18,20,22,27,28]. Furthermore, most experiments are conducted using well-studied species (ex. *E. coli, Bacillus* sp.). Wider use of this technique for bacteria sourced from environmental samples (ex. soil, plants) is, at the moment, rare [29].

In recent years, due to the limitations and negative effects of pesticides and chemical fertilizers, bacteria have been widely studied as potential biocontrol agents and biofertilizers [30]. This search for microbiological alternatives is expected to grow [31,32,33]. Future research in this field will rely heavily on the screening of new and/or yet thoroughly unstudied bacterial species [32,33,34]. Plant associated samples often contain substantial amounts of diverse bacterial species and potentially multiple, difficult to differentiate, strains within a single genus (ex. *Pseudomonas* spp.) [35,36,37,38,39,40]. In the field of plant-associated bacteriology, using SERS for the differentiation and identification of bacteria would likely prove to be highly beneficial and efficient [16,22,29,30,41], as different species and even different strains of the same species may have vastly different effects on plant growth [42].

In this study we aimed to determine whether genetically highly homologous representative endophytic bacterial species from two oak trees from the same site could be effectively distinguished using SERS as an analytical tool in addition to other morphological, physiological and biochemical tests, to help in future biofertilizer research. Also, we aimed to produce specific SERS spectra for different oak-associated *Pantoea*, *Pseudomonas* and *Paenibacillus* isolates for public library building and future use.

With the help of PCA and DFA techniques, we were able to differentiate tested isolates up to strain level based on their SERS spectra. It is noteworthy, that the spectral characteristics of plant-associated bacteria homologous with *Pseudomonas azotoformans* and *Paenibacillus tundrae* species have not yet been described using the SERS method. This SERS-based protocol can be seen as an alternative, cost-effective and fast method for differentiating, identifying and characterizing different types of plant-associated bacteria and promote research in this and associated fields.

## 2. Materials and Methods

Eighteen bacterial samples isolated from two English oaks (*Quercus robur*) were chosen for this study from our previously created library [35], 12 isolates from oak α and six from oak β. Both trees are from the same site, located in Lithuania (Table 1).

### 2.1. Morphological, Physiological and Biochemical Analysis

Morphological, physiological and biochemistry tests were done in triplicates using fresh colonies, grown on low salt lysogeny broth (LB) agarized medium (pH of 7.2 throughout the experiments) (Duchefa Biochemie, Haarlem, the Netherlands) each time. Bacteria were grown at 25 °C. All media were autoclaved prior to use at 121 °C for 15 min. Aseptic techniques were employed throughout the experiments.

#### 2.1.1. Colony Morphology

Colony morphology was observed. Colony form, elevation, margin, color, opacity, smoothness, consistency and overall appearance on LB medium after 2 days of incubation were determined. Additionally, bacteria samples from overnight liquid LB cultures were visualized using 0.1% Gentian violet dye under 10,000× magnification. Bacteria shape, arrangement and size (average from three biological replicates and 10 technical replicates each) were observed.

#### 2.1.2. Biofilm Formation

Bacterial ability to form biofilms was tested. A modified tissue culture plate method was used [43]. Bacteria were grown overnight in liquid LB. The next day 2 µL of this suspension was pipetted into a sterile flat-bottomed 96-well polystyrene tissue culture plate, then each cell was filled with 198 µL of LB medium supplemented with 1% glucose. 200 µL of LB medium supplemented with 1% glucose was used as control. The plate was incubated overnight. After incubation, the plate was washed three times in a new container of sterile water each time. Then the plate was left to air dry. Subsequently, the biofilm layer was dyed using 0.1% Gentian violet solution for 15 min. Afterward, the plate was washed and dried as previously described. After the fixation step, the biofilm layer was solubilized in ethanol (95%) for 30 min. Optical density (OD) was measured using Synergy HT Multi-Mode Microplate Reader (Biotek Instruments Inc., Bad Friedrichshall, Germany) at 630 nm (95% ethanol as control). Optical density cut-off (ODc) was calculated: ODc = average OD of control + 3 times the standard deviation of control. Biofilm formation capabilities were evaluated: weak biofilm ~ODc, moderate—2–4 ODc, strong biofilm—more than 4 ODc.

#### 2.1.3. Carbohydrate Use

A modified phenol red test was used to determine how and which carbohydrates could these isolates use as a carbon source [44]. Lactose (L), fructose (F) (Merck, Darmstadt, Germany), maltose (M) (Avantor, Radnor, PA, USA), sucrose (Su) and glucose (G) (Duchefa Biochemie) were tested. LB liquid medium supplemented with 1% of one selected carbohydrate in each tube and 0.0018% of phenol red dye (Merck) was used. Carbohydrate solutions were filter sterilized and added to the medium after autoclaving. To check for gas production, an upside-down Durham tube was placed in each test tube. The tubes were then inoculated, gently mixed and incubated overnight in a stationary position. This allowed for the positive identification of isolates capable of anaerobically fermenting tested carbohydrates. To discern whether the bacteria were capable of aerobic use of carbohydrates, samples were also placed in a thermal shaker overnight. In both cases, color changes from red to yellow were observed. Bubbles in Durham tubes were indicative of gas production and color changes (from red to yellow) indicate a pH change due to acid production, hence the capacity for carbohydrate use.

#### 2.1.4. Antibiotic Susceptibility

Bacterial susceptibility to various antibiotics was determined by using a modified Kirby-Bauer disk diffusion test [45]. Ampicillin (AM), cefotaxime (CTX), chloramphenicol (C), streptomycin (STP), ticarcillin (TIC) (Duchefa Biochemie) and kanamycin (K) (Panpharma, La Selle-en-Luitré, France) were used. Bacteria were grown overnight in liquid LB medium. The next day the bacterial suspension was adjusted to approximately 1.5 × 10^8^ cfu/mL. The suspension was cross-streaked on Mueller-Hinton agar (Condalab, Madrid, Spain) using sterile cotton swabs. Then sterile 0.5 mm paper disks were placed on top (6 disks per Ø9 cm plate, equally spaced). Filter sterilized antibiotic solutions were then pipetted onto the disks so that each disk contained a desired amount of antibiotics (10 µg of AM and STP, 30 µg of CTX, C, K and 75 µg of TIC per disk). The plates were incubated overnight in the dark. Inhibition zones were measured the next day and bacterial susceptibility was determined using antibiotic susceptibility charts [46,47,48].

### 2.2. SERS Analysis

We used SERS for bacterial vibrational fingerprinting. This method allowed us to sort bacterial isolates into groups using their vibrational patterns and to tentatively ascertain their molecular composition.

#### 2.2.1. Experimental Set Up for SERS Spectra Acquisition

SERS spectra were recorded using Raman spectrometer (NTEGRA Spectra, NT-MDT Inc., Moscow, Russia) in an “upright” configuration with 532 nm laser as the excitation source. All spectra were calibrated to the first-order silicon longitudinal-optical (LO) phonon peak at 520 cm^−1^. The instrument is equipped with 2 mW power at the sample, a 100× objective (NA: 0.7). A thermoelectrically cooled (−60 °C) charge-coupled device (CCD) was used as a detector. The spectral resolution was 1.1 cm^−1^.

#### 2.2.2. SERS Substrate Preparation

The preparation of SERS substrates was based on direct silver ions reduction by elemental silicon [49]. Silicon slides were cut into small pieces (1.5 × 1.5 cm). Then they were polished (2 min) with glass paper to rough up the silicon surface. Such prepared slides with etched 100 mm deep wells were washed with pure ethanol, then dried under nitrogen flow and kept in closed Petri dishes until use.

Preprepared HF (24%) and AgNO_3_ (20 mM) solutions were mixed in a ratio of 1:1 *v/v*. Polished silicon slides were immersed in the reaction mixture for 2 s, then immediately transferred to a container with distilled water (dH_2_O) and finally dried under nitrogen flow. The dried substrate slides were immediately used for SERS spectra measurements.

#### 2.2.3. Bacteria Sample Preparation for SERS

Bacteria from our library were transferred using a plastic loop into liquid LB. Overnight cultures (~10^6^ cfu/mL) were centrifuged at 3500× *g* and washed 3 times with 0.9% NaCl solution. After the last wash bacteria were placed in 200 µL of 0.9% NaCl [12,24]. Using a sterile pipette, 20 µL of the suspension was then placed on the substrate silicon slide and immediately transferred to the Raman microscope for data acquisition. It should be noted that the SERS spectra measurements were performed in the presence of bacteria in liquid suspension and by scanning the sample, thus reducing the thermal effect of the laser on the bacteria, i.e., scanning live samples [50].

#### 2.2.4. SERS Spectra Acquisition

To ensure the reproducibility of the SERS spectra, 50 spectra from each bacterium were obtained from the suspension drop on the SERS substrate. The single spectrum was acquired as a summary spectrum by scanning 100 × 100 micron area during the SERS spectra acquisition to optimize the Raman signal strength. The bacterial spectra dataset was collected from the 50 randomly selected spots in the sample. The acquisition time of Raman scattering signal was 20 s. From the 50 acquired spectra, 16 spectra, based on their signal-to-noise ratio, were selected for processing. The resulting SERS spectra were analyzed and edited using Nova 1.1.0.1840 (NT-MDT Inc., Moscow, Russia) and SpectraGryph 1.2.14 software (Dr. Friedrich Menges, Obersdorf, Germany) with cropping to 600–1800 cm^−1^, removal of background fluorescence, normalization to the intensity of maximum amplitude, baseline correction—5% coarseness [51].

Vibrational bands were noted (peak finding threshold—0.5%, position tolerance—0.4%) and tentative band assignments were determined based on literature sources.

#### 2.2.5. Multivariate Cluster Analyses

Bacterial differentiation was done based on cluster map methodology using PCA and DFA with Raman processing software [52] in the MATLAB (2012) environment (MathWorks, Inc., Natick, MA, USA). PCA was employed for this study to highlight the variability existing in the spectral data set. The reference spectrum for a single isolate used for DFA was produced as an average spectrum of the 16 experimentally acquired spectra. In DFA a leave-one-out cross-validation method was used.

## 3. Results

### 3.1. Morphological, Physiological and Biochemical Analysis

**Figure 1:**
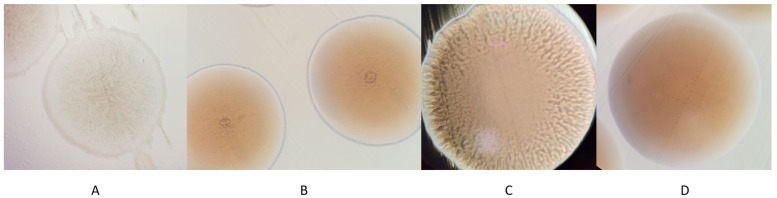
Colony morphology of 4 morphotypes (**A**–**D**) under 40× magnification on LB agar medium after 2 days incubation.

Eighteen bacterial isolates were studied. Twelve from oak α and six from oak β. Previously *16S rRNA* gene fragments were successfully sequenced for all the isolates [35]. All of them were identified to genus level (Table 2). Colony morphology and DNA sequencing results allowed to presumptively divide 18 isolates into four morphotypes, identified as A–D (Table 2, Figure 1). Morphotypes A and D were isolated from both trees, while morphotypes B and C were only isolated from different trees each. NCBI Blast results showed that morphotype A was from the *Paenibacillus* genus and was closely related to *Paenibacillus tundrae*, morphotype B was closely related to *Pantoea agglomerans*, morphotype C—to *Pseudomonas brenneri*/*proteolytica* and morphotype D—to *Pseudomonas azotoformans*.

Bacteria were all similar in diameter—0.28–0.45 µm. Results from morphological, physiological and biochemistry tests also divided the isolates into four distinct groups that coincided with previously described morphotypes (Table 3).

Morphotype A was sensitive to AM, C and K, and capable of fermenting all the carbohydrates tested. Morphotype B was resistant to TIC and capable of fermenting all the carbohydrates tested. Morphotype C formed biofilms, was sensitive to K and capable of using G as a nutrient. Morphotype D was sensitive to K and capable of using G as a nutrient.

### 3.2. SERS Analysis

#### 3.2.1. Structural Analysis Based on SERS Spectra

Eight isolates were selected for SERS analysis. As *Pseudomonas* sp. are difficult to differentiate to species level via *16S rRNA* gene sequencing and since isolates 24 and 29 are of the same origin, we treated them as equal. Thus, for further analysis, isolate 24 and isolates 37 and 49 from the pseudomonad group were chosen. Isolates 37 and 49 were highly homologous and from different sources. Isolates 33.1 and 35 were selected from the *Paenibacillus* sp. group, because based on genetic tests and additional experiments, they were identical, but of different origins. Moreover, isolates 27, 30 and 34, representing *Pantoea agglomerans*, were selected for vibrational analysis. They were all sourced from the same tree, however, they exhibited differences in plant hormone, indole-3-acetic acid (IAA), production in previous studies [35]. To determine the efficacy of the proposed bacterial differentiation methodology, the focus was put on within-group differences of isolates 33.1/35, 27/30/34 and 37/49.

**Figure 2:**
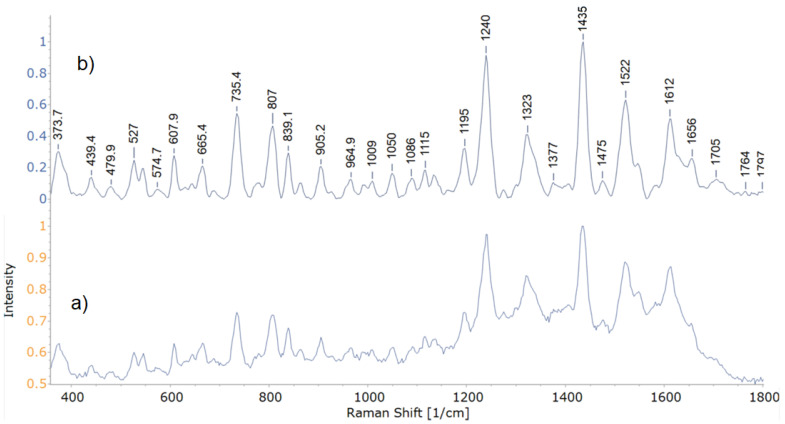
Representative experimental (**a**) and preprocessed (**b**) spectra of isolate 27.

**Figure 3:**
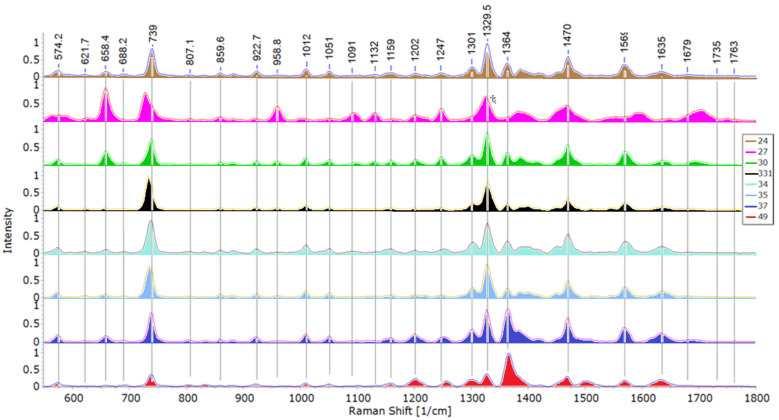
Stacked mean spectra of 8 bacterial isolates with peak values for the most notable bands.

Band peaks and their respective intensities are used to sort bacteria in relation to one another [7,29,50]. Representative spectra acquired during this study are shown in Figure 2. The stacked mean spectra of all 8 isolates are shown in Figure 3.

As mentioned previously, peaks in the SERS spectra are linked with functional groups [6,7,20,22,26]. These groups represent components of bacterial cells [8,17], most often either extracellular polymers [17] or more likely degradation metabolites [50,53] or parts of the outer membrane in Gram negative bacteria [13]. We present tentative SERS spectra band assignments in Table 4.

Minor Raman shifts in various references seen in Table 4 are due to methodological variations [7,15,17] as well as indicative of molecular differences [50], facilitating successful differentiation.

**Figure 4:**
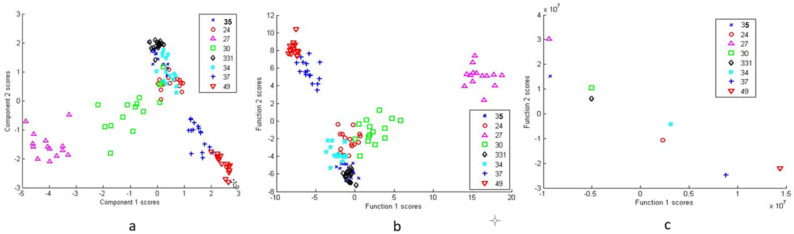
Principal component analysis (PCA) score plot for all 8 isolates (**a**), discriminant function analysis (DFA) for all eight isolates based on PCA scores (**b**) and DFA of all eight isolates using raw spectral data (**c**).

As can be seen from Figure 4a,b, isolate 27 diverged greatly from other tested isolates. It exhibits several peaks, that weren’t observed in other test subjects (peaks at 563, 1005, 1592, 1647, 1703 and 1750 cm^−1^). While peaks at 563, 1005, 1592 and 1647 cm^−1^, are likely indicative of a shift, bands in the 1700 cm^−1^ range, linked with C=O deformation, are wholly unique to this isolate. Isolates 30 and 34 from *Pantoea agglomerans* group didn’t show such differences, however, they diverged by the absence of peaks at 688, 858 and 958 cm^−1^. Additionally, isolate 30 exhibited a peak at 1131 cm^−1^, related to deformations of C–C, C–N in carbohydrates or =C–C= in lipids, alongside isolate 27, while this peak was absent from the spectra of isolate 34.

Isolates 33.1 and 35 from *Paenibacillus* sp. group exhibited similarities in their spectra and were grouped close in PCA and DFA score maps (Figures 4a and 6a). However, as can be seen by their raw data DFA (Figure 6b), there were differences. Isolate 33.1 has a notable peak at 1398 cm^−1^, linked with COO– or CH_3_ deformations, and lacks notable peaks at 688, 804 and 1383 cm^−1^, while isolate 35 has a notable band at 1116 cm^−1^, potentially related to Trp. The peak at 1398 cm^−1^ is likely related to peaks at ~1388 cm^−1^, indicating a shift, rather than an absence. Moreover, the peak at 1116 cm^−1^ is unique for isolate 35, since isolate 33.1 doesn’t exhibit a peak related to that area.

Isolate 24 exhibits a peak at 1421 cm^−1^, linked with lipids or carbohydrates, that the other two isolates, 37 and 49, from the pseudomonads group lack, while missing a peak at 1383 cm^−1^. Isolate 37 has peaks at 966 cm^−1^ and 1119 cm^−1^ and lacks a peak at 1679 cm^−1^ (all linked with proteins), that isolate 49 exhibits. The peak at 966 cm^−1^ can potentially be related to the peak at ~958 cm^−1^, both, based on past studies, linked with C–N deformations. Furthermore, isolate 49 doesn’t show notable peaks at 622, 858 or 882 cm^−1^, while exhibiting a peak at 1501 cm^−1^, which according to our findings, is linked with various organic compounds, carotenoids among them. Most of these peaks are unique to isolate 49, except for the peak near 1500 cm^−1^, which may be a shift from the carotenoid band at ~1510 cm^−1^.

#### 3.2.2. Differentiation via Multivariate Cluster Analyses

Cluster analysis methods were used for bacterial differentiation. Figure 4a shows the PCA scatter plot of the eight bacterial isolates based on 14 principal components (PCs) accounting for 95.1% of the variance. Here, the scatter plot presents the ability of the SERS spectral analysis to differentiate among the different types of bacteria.

SERS data of the tested isolates were also classified using DFA. Eleven PC results were used as independent input variables in DFA (Figure 4b), which further reduced the spectral dimension, however, the groupings remained similar to those of the PCA. Furthermore, DFA from raw spectra data is presented in Figure 4c. Each isolate is sorted out as an individual, but close relationships within and between groups are noticeable. Two DF scores were calculated for each spectrum for the three bacterial cell types.

**Figure 5:**
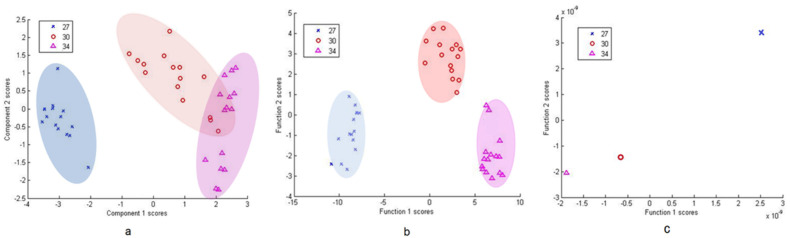
PCA of *Pantoea agglomerans* group (27, 30, 34) (**a**), DFA from PC scores (**b**) and DFA from raw data (**c**). Isolate 27 is individually grouped in all 3 analyses, while DFA facilitates clear separation between all the tested isolates.

**Figure 6:**
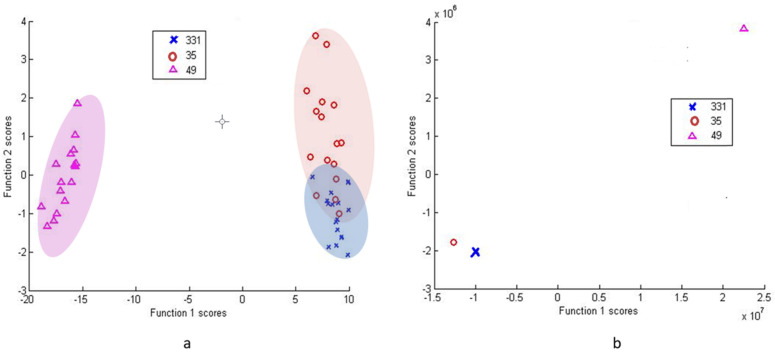
DFA of *Paenibacillus tundrae* group (33.1 and 35) with *Pseudomonas* isolate 49 as an outlier, as 33.1/35 are highly similar. While DFA from PCA scores shows differentiation with overlaps (**a**), DFA from raw data (**b**), while still demonstrating homology, is able to separate isolates 33.1 and 35.

**Figure 7:**
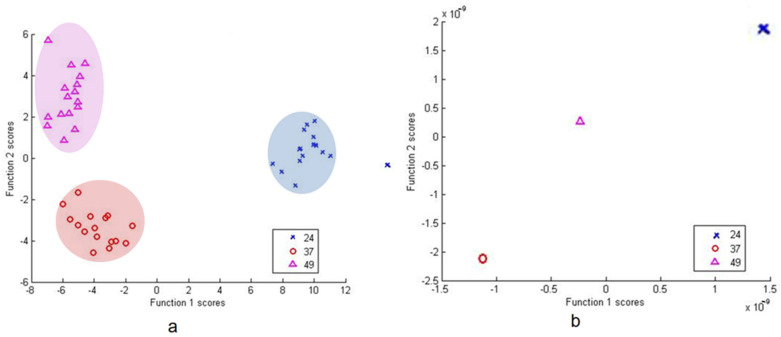
DFA based on PCA scores (**a**) and raw data (**b**) of *Pseudomonas* sp. group (37, 49 and 24).

For in-depth within-group separation DFA was used further (Figure 5, Figure 6 and Figure 7). PCA of group 27/30/34 shows a clear disassociation of isolate 27 (Figure 5a). DFA from PC scores of isolates 34, 30 and 27 was able to correctly classify 100% of each group’s subjects (Figure 5b). DFA based on raw data with cross-validation between isolates 30 and 27 was able to correctly classify 100% of subjects in the groups and DFA based on raw data with cross-validation between isolate 30 and 34 was able to correctly classify 100% of subjects in the group 34 and 93.8% of subjects in the group 30 (Figure 5c). Thus, the results show that isolate 27 can be effectively differentiated from the other two isolates in the group.

DFA based on PC scores with cross-validation between isolates 33.1 and 35 was able to correctly classify 87.5% of the group 33.1 subjects and 79.1% of the group 35 subjects (Figure 6a). The DFA using raw data with cross-validation between 33.1 and 35 was able to correctly classify 100% of subjects in both groups (Figure 6b). 

DFA based on PC scores and from raw data with cross-validation between isolate 37, 49 and 24 was able to correctly classify 100% of subjects in all the groups (Figure 7).

## 4. Discussion

This study showcases that SERS coupled with multivariate cluster analyses can serve as an effective means to achieve bacterial differentiation in plant-associated species, as opposed to standard *16S rRNA* gene sequencing and additional antibiotic susceptibility, carbohydrate use, biofilm formation and phenotyping tests.

Additional tests performed during this experiment were able to account for genus level separation. Colony morphology and antibiotic susceptibility tests were more effective than biofilm formation and carbohydrate use studies. Antibiotic susceptibility is considered a strain-level response [80]. Bacterial strains also have been shown to be able to adapt to utilizing new carbohydrates through mutation [81]. *Pseudomonas* sp. are known biofilm producers [82] and indeed biofilm formation test was able to separate two pseudomonads capable of this. Biofilms are extracellular structures often containing polysaccharides as well as other compounds [3], thus it is possible that evidence of them may be noted in the SERS data, as was shown previously with several species [83,84,85]. However, it’s worth noting that the methodology chosen in this study isn’t ideal for yielding data on biofilms created by isolate 24.

While comparable studies showed that SERS works for pathogenic, medicine and food related, strains [1,8,9,20], based on available information, the usefulness of this technique was not widely studied for plant-associated bacteria [29], or even more specifically for endophytes.

The complexity of the SERS spectrum makes interpretation of the data challenging. Statistical procedures or chemometric multivariate analyses are designed to improve the use and interpretation of experimental data [22]. Chemometrics is defined as a mathematical method used to extract useful information from measured physicochemical data [86].

In this study PCA and DFA, multivariate data analysis techniques, were applied to the SERS spectral data. PCA is one of the multidimensional descriptive methods in chemometrics, particularly fitting for the study of spectral data. This technique provides a synthetic image by presenting factor maps (2D or 3D), in which each spectrum is represented by a dot. The primary variables are replaced with synthetic ones (principal components), which contain all the information [15,87], thus interpreting PCA maps makes it relatively easier to understand the structure of the spectral data [88].

DFA allows for the rapid sorting/grouping of unknown spectra based on between-group variability while minimizing within-group variability [6,52]. It also facilitates immediate validation of spectral reproducibility, as very similar spectra should have very similar discriminant function scores and should consequently be closely grouped in DFA. All in all, DFA and PCA are similar in that they both reduce the dimension of the data, but DFA provides better separation between groups of experimental data in comparison to PCA. Additionally, while DFA may need a certain level of a priori knowledge about the spectra, PCA is used to examine raw data [87].

PCA has been successfully used for SERS spectra analysis in other bacterial differentiation studies [1,29,69,89]. DFA has been used as well, however, less often [52,63,77].

It is noteworthy, that the label-free SERS spectra acquisition protocol presented in this study is an easily replicated approach for procuring bacterial spectra, as bacteria are in an aqueous solution, requiring minimal preparation. Most often comparable procedures either use colloidal solutions or dry out the sample, hence facing difficulties with thermal damage [9,15], which may affect spectra acquisition, as often carbon associated peaks arise in the biologically relevant range [9].

Based on current knowledge, the methodology used in this study likely showcases the metabolic degradation of the tested bacteria, as they are in a no-nutrient environment (salt solution). Nevertheless, as this is linked with specific enzymes each strain may produce and unique metabolic pathways, it too ultimately relates to biochemical differences and thus strain-specific differentiation [50,53]. Another recent study demonstrates that SERS peaks may also derive from the constituents of bacterial outer membrane (Gram-) [13].

Moreover, nucleotides are rarely seen in extracellular regions, but notable bands for them have been found in various studies [9,10,15,26,29]. Bands in the same regions have been observed in this study as well. For example, an intense peak at ~730 cm^−1^ is commonly assigned to adenine-type compounds [90]. Furthermore, adenine molecules are part of adenosine triphosphate (ATP), which bacteria use for energy, hence it is possible that their degradation metabolites would end up outside of the bacterial cells, as have been shown with *E. coli* placed in starvation mode [91] and other studies [50,92].

In this study, bacteria from three different genera were examined. Although certain similarities can be observed in all the tested subjects, i.e., the aforementioned adenosine band at ~730 cm^−1^, unique vibrational signatures for all of them were successfully obtained. Similar results have been presented previously, whereby *E. coli*, *Listeria monocytogenes* and *B. subtilis* strains were shown to have different SERS spectra [11,67,89,93]. Furthermore, Premasiri et al. state that mutations and even genealogy may be observed in their SERS data [11].

While the exact nature of the bacterial Raman/SERS bands is difficult to assign, without mutant bacteria studies, due to peak overlap and minor shifts, bacterial differentiation is still possible [7,15].

Bacterial strains genetically homologous with *Pantoea agglomerans* have been investigated in previous Raman studies [94]. *P. agglomerans* vibrational fingerprint reported by Guicheteau et al. resembles those obtained in this study. There were differences though, for example, the peak at 536 cm^−1^, that the authors attributed to cysteine, asparagine or glutamine, was found shifted in the spectra of isolate 27, and absent from the other two tested isolates. Moreover, isolate 34 didn’t have a notable peak at 958 cm^−1^. Several peaks demonstrated shifts (ex. at ~1004, 1142, 1544 cm^−1^), while others are absent in the spectra of the strain tested in the cited work [94]. Furthermore, other species from the *Pantoea* genus have been studied as well [30,77]. A study on IAA-producing *Pantoea* sp. has shown that some notable bands are produced by carotenoids, notably, bands at 1002, 1158 and 1520 cm^−1^, of which analogs were found in our research as well (~1005, 1159, 1570 cm^−1^). Additionally, authors in this study discuss the possibility that the IAA production capacity of this strain may have also been observable, through Trp peaks, as IAA and Trp have similar chemical structures (in fact Trp is a precursor to IAA) [95]. Several Trp associated peaks have been noted in our research as well. This too may potentially be linked to IAA production [35].

The most widely researched *Pseudomonas* species are *P. aeruginosa* and *P. fluorescens*. To our knowledge, pseudomonads homologous to those analyzed in this work, have not yet been characterized using Raman techniques. However, data on *P. aeruginosa*, have shown some similarities to the pseudomonad spectra examined in this work. Spectra reported by Yang et al. are similar, as they share some peaks with very minor shifts (ex. 655, 730, 920, 1328, 1470 cm^−1^). A peak near 854 cm^−1^ (related to G) was not observed in isolate 49, while peaks at 957 and 1091 cm^−1^, linked with hypoxanthine, A, G and guanosine, were only noted in isolate 24. Furthermore, authors report peaks at 518, 1219 and 1528 cm^−1^, which were not obtained in this study [63]. Another study on *P. aeruginosa* also demonstrates peaks, that are comparable to those found in this study [70]. However, most of them are expressed differently in different isolates, peaks at ~690 and 832 cm^−1^ (related to G, C and C-O-C stretching according to the authors) are observed only in the data of isolates 37 and 49, while peaks near 958 and 1421 cm^−1^ (linked with phospholipids or C-O deformations) have only been noted in isolate 24. A study done on *P. fluorescens* resulted in the SERS peak at 1495 cm^−1^ [78]. During our research, this peak was found to be one of the discerning factors of isolate 49, homologous with *P. azotoformans*. This peak has been linked with lipids, proteins and carotenoids (see Table 4).

Plant-associated *Paenibacillus validus* has exhibited many of the same peaks, as those reported in our study for isolates 35 and 33.1 (958, 1009, 1158, 1363, 1470, 1511, 1570, 1680 cm^−1^) [29]. Nonetheless, the article didn’t enlighten on the origins of peaks that discerned the *Paenibacillus* sp. isolates in our experiment (1398 and 1116 cm^−1^).

## 5. Conclusions

A rapid SERS strategy for bacteria differentiation was successfully established by using low-cost AgNP/Si substrates, where other methods (biochemical and genetic testing) have failed. It was found that AgNP 3D film on Si surface was interacting with the bacteria, resulting in strong and reproducible SERS spectra. Surface enhanced Raman spectroscopy coupled with advanced statistical techniques (PCA and DFA) were used to discriminate between different plant bacterial strains of *Paenibacillus*, *Pseudomonas* and *Pantoea* genera by probing the molecular components of their cells. This is the first time, SERS peaks characteristic to bacteria closely related to *Pseudomonas azotoformans* and *P. brenneri/proteolytica* have been obtained. Moreover, so far as we were able to determine, this is one of the first studies on the SERS spectra characteristics of *Paenibacillus* sp. This work progresses the current knowledge of bio-spectroscopy and may help with the introduction of SERS-based bacterial identification technique as a standard method of analysis in plant-associated bacteriology.

## Figures and Tables

**Table 1:** Tree locations and their associated isolate identification numbers.

Tree	Site (GPS Coordinates)	Isolate Identification No.
Alfa (α)	55.829832, 26.217380	21, 27, 30, 32, 33, 33.1,34, 36, 40, 46.1, 46.2, 49
Bravo (β)	55.8301132, 26.2168633	24, 29, 35, 37, 47.1, 47.2

**Table 2:** Colony morphology and *16S rRNA* sequencing results of 18 bacterial isolates and their corresponding morphotypes (in part based on [35]).

Isolate Identification Code	Colony Morphology	Morphotype	Closest NCBI Match, Accession No., % Identity
21, 33.1, 35	Colonies are circular, flat with a slightly undulate margin, smooth and glistening, off-white with a grey bull’s eye in the center, translucent and mucoid.	A	*Paenibacillus tundrae* A10b, NR_044525.1, 99.32–99.46%
27, 30, 34	Colonies are circular, flat, cream colored, translucent, smooth and glistening, butyrous, the margin is entire. Changes LB agar medium color to bright yellow. A small spindle formation can be observed at the center of the colony with 40× magnification.	B	*Pantoea agglomerans* DSM 3493, NR_041978.1, 99.64–99.97%
24, 29	Colonies are circular, raised, buff color, glistening and butyrous, the center of the colony is rough, and the edges are smooth, the margin is entire. The colonies change color of LB agar medium to bright yellow.	C	*Pseudomonas brenneri* CFML 97–391, NR_025103.1, 99.86%; *Pseudomonas proteolytica* CMS 64, NR_025588.1, 99.59%
32, 33, 36, 37, 40, 46.1, 46.2, 47.1, 47.2, 49	Colonies are circular, flat, cream colored, translucent, smooth and glistening, butyrous, the margin is entire. Changes LB agar medium color to bright yellow.	D	*Pseudomonas azotoformans* NBRC 12693, NR_113600.1, 99.66–99.79%

**Table 3:** Analyzed properties (size, biofilm formation, carbohydrate use, antibiotic susceptibility) of 18 bacterial isolates used in this study (in bold—isolates selected for further SERS analysis) *.

Morphotype	Isolate Identification No.	Average Diameter ± SD, µm	Biofilm Production	Antibiotic Disk Diffusion Test						Carbohydrate Use				
				AM-10	CTX-30	C-30	K-30	STP-10	TIC-75	L	M	Su	G	F
**A**	21	0.444 ± 0.03	−	S	R	S	S	R	R	Ac	Ac	Ac	Ac	Ac
	**33.1**	0.361 ± 0.039	−	S	R	S	S	R	R	Ac	Ac	Ac	Ac	Ac
	**35**	0.43 ± 0.042	−	S	R	S	S	R	R	Ac	Ac	Ac	Ac	Ac
**B**	**27**	0.324 ± 0.041	−	S	S	S	S	S	R	Ac	Ac	Ac	Ac	Ac
	**30**	0.352 ± 0.04	−	S	S	S	S	S	R	Ac	Ac	Ac	Ac	Ac
	**34**	0.438 ± 0.028	−	S	S	S	S	S	R	Ac	Ac	Ac	Ac	Ac
**C**	**24**	0.326 ± 0.033	moderate	R	R	R	S	R	R	−	−	−	Ac	−
	29	0.284 ± 0.014	moderate	R	R	R	S	R	R	−	−	−	Ac	−
**D**	32	0.366 ± 0.021	−	R	R	R	S	R	R	−	−	−	Ac	−
	33	0.338 ± 0.03	−	R	R	R	S	R	R	−	−	−	Ac	−
	36	0.447 ± 0.047	−	R	R	R	S	R	R	−	−	−	Ac	−
	**37**	0.34 ± 0.023	−	R	R	R	S	R	R	−	−	−	Ac	−
	40	0.427 ± 0.037	−	R	R	R	S	R	R	−	−	−	Ac	−
	46.1	0.426 ± 0.036	−	R	R	R	S	R	R	−	−	−	Ac	−
	46.2	0.338 ± 0.028	−	R	R	R	S	R	R	−	−	−	Ac	−
	47.1	0.43 ± 0.073	−	R	R	R	S	R	R	−	−	−	Ac	−
	47.2	0.45 ± 0.042	−	R	R	R	S	R	R	−	−	−	Ac	−
	**49**	0.405 ± 0.042	−	R	R	R	S	R	R	−	−	−	Ac	−

*—signifies a negative result; R: resistant, S: sensitive, Ac: acid production.

**Table 4:** Tentative assignments of the most notable surface enhanced Raman spectral bands of mean spectra from 8 tested isolates.

Peak Wavenumber, cm^−1^								Tentative Band Assignments *
24	27	30	33.1	34	35	37	49	
	563.38							T, G [10,54]
574.21		573.52	573.01	573.47	572.08	573.63	573.57	Deformation of C=O–C in lipids [55] or Trp [54,56,57] or carbohydrates [58]
621.65	625.66	622.17	623.31	620.93	622.5	622.03		C–C twisting mode of Phe ring [7,10,29,54,56]
658.43	657.8	658.53	657.42	657.71	657.09	657.93	658.28	G, ring breathing mode [9,59,60,61] or amino acids COO–[62]
688.19				689.83	689.18	689.93	692.23	C–S stretch [26,57] or Gly [54]
739.19	727.8	737.96	733.83	737.14	734.21	738.08	737.93	A, glycosidic ring breathing [9,10,15,26,63,64]
807.14	804.25	804.8		805.45	807.23	801.95	804.72	O–P–O [1,26] or C–N stretch [20]
836.42		832.58		831.81		830.52	832.87	O–P–O stretching in T [10,59] or Tyr [29,59]
859.65	858		858	859.14	859.65	858.69		Phosphodiester, deoxyribose related to T [4,10] or Tyr [29]
882.32		879.93		882.2		880.81		T, ring bending [10], stretching of C–N or C–O–N or deformation of C–C–H [65]
922.65	919.72	922.49	920.2	922.4	922.32	922.51	922.53	C–COO– stretch in carbohydrates [26,66]
958.79	959.67	957.92	958.24		958.22			C–N stretching [7,10,29,67] or C–C/C–O stretching in membrane proteins [10]
						966.22		C–N stretch [26] or C=C deformation in G [61]
	1005.6							Phe [7,10,29]
1011.9		1009.1	1009.5	1010	1009.6	1009.5	1009.4	Phe [68,69] or Trp [54,57]
1051	1050.3	1050.6	1050.9	1050.8	1051.1	1050.4	1050	Phenylalanine (the in-plane C–H bending mode) [69] or stretching of C–O/CH_2_–OH in lipids [70]
1091.2	1094.7	1099.2		1089.9				PO_2_^-^ of nucleic acid stretching [10,20,29] or deformation in carbohydrates(C–C, C–O, –COH) [7,65,67]
					1116.4	1119.2		Trp [65]
	1131.1	1129.6						C–N and C–C stretching in carbohydrates [61,69] or =C–C= in unsaturated fatty acids in lipids [16,29]
1159.3	1161.7	1159.8	1158.5	1158.5	1159.6	1158.3	1158.7	C–C/C–N stretching in proteins [10] or carotenoids [15,29]
1202	1201.9	1203	1200.8	1201.5	1201.6	1201.1	1201.7	=C–C= in lipids [69] or aromatic amino acids in proteins [71]
1247.2	1247.4	1247.6	1246.4	1254.8	1250.9	1252.5	1256.9	Amide III [10,26,29,62,65]
1301.4		1301.7		1303.1		1301.6	1300.4	CH_2_ twist in lipids [10,55]
1328.4	1325.2	1327.7	1328.2	1328.5	1328	1327.4	1327.3	A [9,62,63,70,72]
1363.7		1363.3	1363.5	1363.8	1363.8	1364.7	1366.4	Trp [10] or C–H deformation in proteins/COO– deformation [5,73]
	1383	1387.1		1388.8	1386.3			COO– stretching in proteins [66,74] or CH_3_ bending [29]
			1398.8					COO– symmetric stretching [1,75] or deformation of CH_3_ [76]
1421.2								CH_2_ deformation in lipids [50,66,68] or A, G [66,71]
1470.4	1467.8	1469.6	1470	1470	1470.2	1469.6	1468.8	Lipids [9,10] or deformation of C–H in proteins [65,69]
							1501.2	Fatty acids in lipids [5,73] or carotenoids [29] or amino acids [74]
			1511.1		1511.5			Carotenoids [29,77] or Phe [70]
1569.4		1570.5	1570.1	1570.9	1569.9	1569.5	1569.3	Tyr/proteins [69,70] or A/G [78]
	1592.4							Proteins [10] or A/G [1,7] or Tyr [72,79]
1634.5		1634.7	1636.8	1634.5	1635.5	1633.1	1633	Amide I in lipids [10,26,72]
	1647.9							Amide I [69] or T [29,68]
1679.2		1694	1681.1	1684.3	1676.6		1682.8	Amide I [10,62,71,75]
	1703.4							C=O [5,71]
	1750.2							C=O stretching [5,21]

* T: thymine, G: guanine, Trp: tryptophan, Phe: phenylalanine, Gly: glycine, A: adenine, Tyr: tyrosine.

## Data Availability

Detailed data concerning this study is available upon request.
